# Supplementary figures and images for: Potential diagnostic markers and therapeutic targets for DM2 and periodontitis based on bioinformatics analysis
Source: PLoS One. 2025 Apr 2;20(4):e0320061. doi: 10.1371/journal.pone.0320061 (PMC11964240; doi:10.1371/journal.pone.0320061)

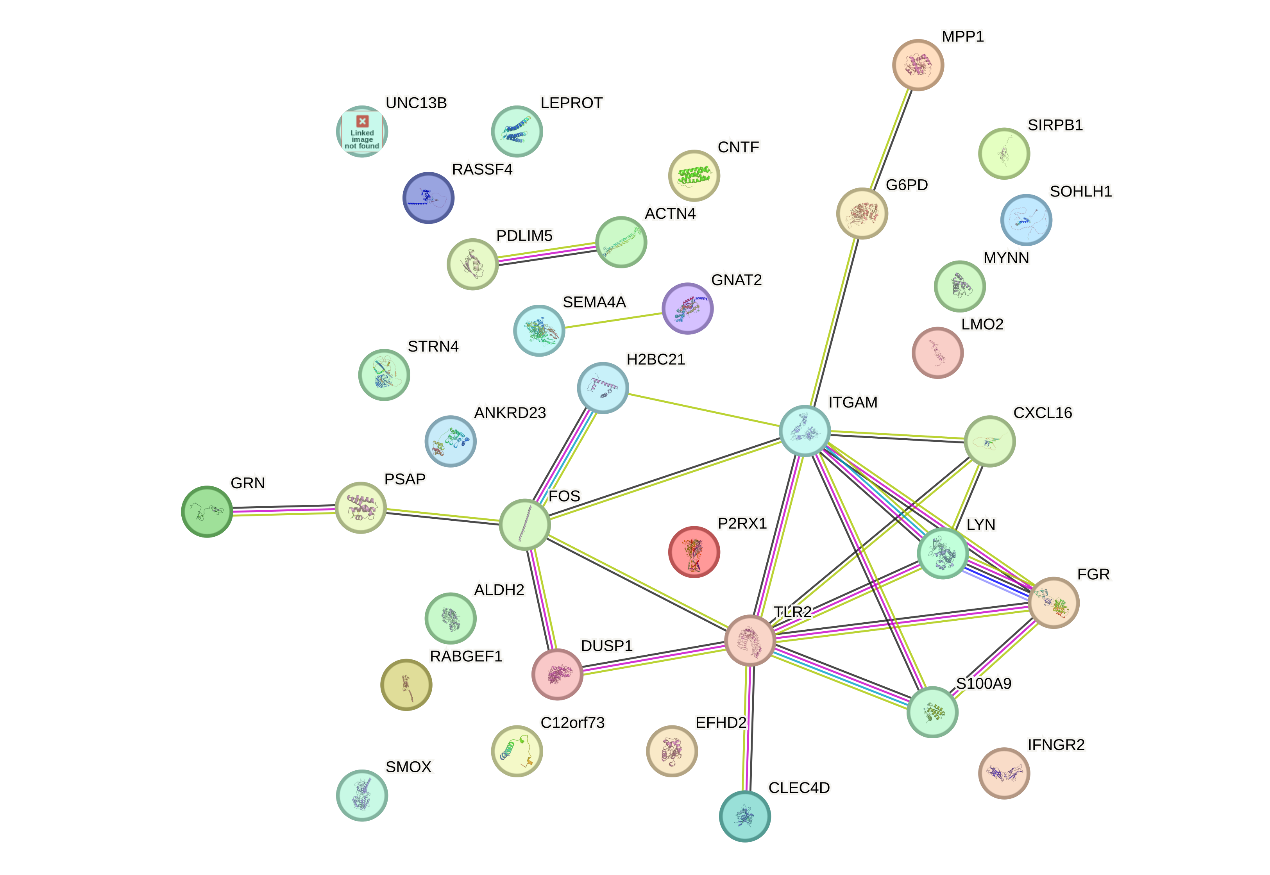


**Supplementary Fig S1**. The PPI network

Supplement: S1 Fig — (DOCX) [file pone.0320061.s001.docx]
