# Supplementary material for: Potential diagnostic markers and therapeutic targets for DM2 and periodontitis based on bioinformatics analysis
Source: PLoS One. 2025 Apr 2;20(4):e0320061. doi: 10.1371/journal.pone.0320061 (PMC11964240; doi:10.1371/journal.pone.0320061)
Supplement: Attachment — Submitted filename: Response to Reviewers.doc [file pone.0320061.s003.doc]

Dear Editor:

I am the author of the manuscript (Identification the hub genes of periodontitis and diabetes by bioinformatics and experiment, ID: PONE-D-23-39620). I have revised the manuscript according to the comments carefully.

Reviewer #1: 1. English expressions need to be edited more careful and more native, in this manuscript, there are some mistakes. For example, there is no period in the last sentence in method of the abstract.

**Reply**: Thank you for your suggestion. I have polished my article.

2. I suggest the authors should add a flowchart in the manuscript to show the process very well.

**Reply**: Thank you for your suggestion. I have added a flowchart in the manuscript.

3. I suggest the authors should elaborate their motivation in the discussion section, as they use several common bioinformatics analysis methods and online tools. What is the novelty and technicality of their work?

**Reply**: Thank you for your suggestion. I have added to the discussion section about our use of this bioinformatics analysis method and online tools.

4.The advancement of interaction prediction research in various fields of computational biology would provide valuable insights into genetic markers and ncRNAs related with DM, such as miRNA-lncRNA interaction prediction. The authors should discuss it as the future direction. Important computational models in these fields should be cited. Some recommended studies are helpful (PMIDs: 36642414, 36924730, 36305458, 34232474, 37525507, 37660567 and 37466194).

**Reply**: Thank you very much for your constructive comments. I have carefully studied the relevant literature research provided by you, which will benefit me a lot in my future research methods and methods. And in my discussion part, I introduce the model of relevant literature for discussion.

5. The authors should carefully check and unify the information of references. Some references lack the information of volume or contain the wrong page number.

**Reply**: Thank you for your suggestion. I have carefully checked and unified the information of references.

6. Literature review is incomplete in the introduction, especially about the research of differentially expressed genes (DEGs) involvement in periodontitis by computational tools or bioinformatic analysis. I suggest the authors to discuss the recent updates in the related field. Different feature weight calculation methods within a single algorithm result in divergent rankings of DEGs. I think we need consistency and comparable results.

**Reply**: Thank you for your suggestion. I quite agree with you. In fact, we used Degree, EPC, MNC and MCC algorithms to score DEGS, and finally screened out candidate genes conforming to the above algorithms. This approach is to evaluate the role of DEGS in diseases more objectively. Just like this article (PMID: 36960398).

7. Materials and methods section is relatively simple because of no detail about the analysis. The analysis methods and statistical parameters must be clearly emphasized (i.e, reasons for selecting the algorithms used, threshold values used in statistical analysis, etc.).

**Reply**: Thank you for your suggestion. In the part of materials and methods, I have added the specific process and parameters of relevant algorithms in detail.

Reviewer #2: 1. What do “LPS” and “LAPTM5, RAC2, LYN”represent in the Abstract section? What are their full names? When first mentioned, the author should provide their full names to help readers better understand.

**Reply**: Thank you for your suggestion. I have provided their full names in the abstract.

2.The authors should add a flowchart in the manuscript to show the process very well.

**Reply**: Thank you for your suggestion. I have added a flowchart in the manuscript.

3.The description of the result is quite simple and too short, especially the descriptions of “Identification of Common DEGs” and “The analysis of immune infiltration”. The authors should add some necessary sentences to describe these results.

**Reply**: Thank you for your suggestion. I have provided as detailed a description of the relevant results as possible in the Result part.

4.The labeling of Figures in the paper is quite small. The contents within the figures are unclear. The author needs to carefully revise and modify the figure.

**Reply**: Thank you for your suggestion. I have enlarged the words on my graphics as much as possible according to your suggestion.

5.The discussion of the deficiencies in current research is quite poor. The authors should discuss it as the future direction. ODE-based theoretical modeling studies on gene/protein signaling networks have been equally important for the study of understanding regulatory mechanisms and finding potential therapeutic targets in diseases (PMID: 35958114, https://doi.org/10.1016/j.chaos.2023.114328, and https://doi.org/10.1103/PhysRevE.108.064412). Would it be possible to discuss and cite these studies in conjunction with the conclusions of this paper?

**Reply**: Thank you very much for your constructive comments. I have carefully studied the relevant literature research provided by you. And added the discussion as the future direction.

6.Besides, the advancement of interaction prediction research in various fields of computational biology would provide valuable insights into genetic markers and related diseases. Important computational models in these fields should be discussed and cited. Some recommended studies are helpful (PMIDs: 36584603, 35817399, 36305458).

**Reply**: Thank you very much for your constructive comments. I have carefully studied the relevant literature research provided by you, which will benefit me a lot in my future research methods and methods. And in my discussion part, I introduce the model of relevant literature for discussion.

7.The authors should carefully check and unify the information of references. Some references lack the information of page number, such as refs [7] and [30].

**Reply**: Thank you for your suggestion. I have checked and unified the information of references

Best wishes

Luorong
