# Supplementary material for: Potential diagnostic markers and therapeutic targets for DM2 and periodontitis based on bioinformatics analysis
Source: PLoS One. 2025 Apr 2;20(4):e0320061. doi: 10.1371/journal.pone.0320061 (PMC11964240; doi:10.1371/journal.pone.0320061)
Supplement: Attachment — Submitted filename: ReviewerReply.docx [file pone.0320061.s005.docx]

**Reviewer #1:** The author has already addressed all of the issues. I think the manuscript can be published in PLOS ONE.

**Reply**: Thank you for your recognition.

**Reviewer #3:** Thank you to the authors for the considerable amount of work done in the study "Identification of the hub genes of periodontitis and diabetes by bioinformatics and experiment," as well as for addressing some of the reviewers' comments during the revision process. However, from my personal perspective, I find it difficult to agree with the authors' results and conclusions. The main reasons are as follows:
The authors used the dataset GSE7014, which includes 10 DM1 biopsies, 20 DM2 biopsies, and 6 normal individuals' biopsies of skeletal muscle. The GSE16134 database includes a total of 120 patients undergoing periodontal surgery, each contributing with a minimum of two interproximal gingival papillae. This results in two major flaws for the study. Firstly, Type 1 diabetes and Type 2 diabetes are diseases with completely different genetic and pathological mechanisms, making it entirely unacceptable to combine them. Secondly, there is a significant difference in gene expression between the interproximal gingival papillae and skeletal muscle tissues in the two datasets. Therefore, I regret to say that I cannot agree with your analysis results and conclusions.

**Reply**: Thank you very much for your valuable advice on my research. I rearranged our entire research program with your advice. In view of the “Type 1 diabetes and Type 2 diabetes are diseases with completely different genetic and pathological mechanisms”, I agreed that it was wrong to mix the two together. Therefore, I focus on DM2 as the main analysis object and PD analysis. Furthermore, for the point of “there is a significant difference in gene expression between the interproximal gingival papillae and skeletal muscle tissues in the two datasets”, I re-select GSE6751 (PD) and GSE15932 (DM2). The samples for these two data sets are from Peripheral Blood Mononuclear Cells (PBMC) samples. Through the above adjustments, I found more convincing results and conclusions than before. Thank you very much for your suggestions, which have laid a good foundation for my future work.

**Reviewer #4:** The manuscript titled "Identification of the hub genes of periodontitis and diabetes by bioinformatics and experiment" addresses an interesting intersection between periodontitis and diabetes mellitus, suggesting that shared molecular mechanisms could be explored via bioinformatics to identify key genes (LAPTM5, RAC2, and LYN). While the authors have provided a detailed bioinformatic analysis and experimental validation, there are significant concerns regarding the novelty and depth of the study: The idea of identifying shared pathways between periodontitis and diabetes using bioinformatics is not entirely novel. Similar studies exist that delve into shared molecular mechanisms and immune responses in various diseases, including periodontitis and diabetes. The manuscript does not adequately highlight how its findings significantly advance the existing body of knowledge beyond what has been previously published. While the study follows a well-known bioinformatic pipeline (DEGs identification, functional analysis, and protein-protein interaction network), it lacks innovation in methodology. The application of widely-used datasets (e.g., GSE7014 and GSE16134) without integrating more recent or diverse datasets from single-cell RNA sequencing (scRNA-seq) or other omics approaches limits the potential impact of the findings. Furthermore, the study's use of in vitro validation, while helpful, does not explore functional assays beyond qPCR, and the validation itself remains quite basic. The study relies on standard bioinformatic tools, but statistical robustness should be further scrutinized. For example, the selection of hub genes might benefit from additional cross-validation or more advanced machine learning techniques, which would add weight to the findings.
The authors have not integrated the latest available data on single-cell RNA sequencing for periodontitis or diabetes. Recent studies have shown the value of scRNA-seq in understanding cellular heterogeneity in diseases. The omission of these newer datasets weakens the study's relevance in the current scientific landscape.
Given these limitations, I would recommend rejecting the paper at this stage. The manuscript could benefit from incorporating more novel datasets, improving statistical validation, and considering recent developments in the field such as scRNA-seq for a more comprehensive analysis. Additionally, a more thorough discussion of how the results compare to and advance the current understanding of periodontitis-diabetes interactions would be necessary for future revisions.

**Reply:** Thank you for your valuable comments on my research. I re-adjusted my whole research according to the reviewer's opinion. Through this adjustment, I found a potential biomarker for type 2 diabetes and periodontitis -S100A9. This finding is the first in the current study to propose S100A9 as a biomarker for both. The correlation was verified by PCR and WB experiments. Although I know my research still has shortcomings, please give me a chance, so that I can continue to have confidence in the follow-up experiment. Thank you very much for your advice. I think it is a very important experience in my academic career.

**Reviewer #5:** The manuscript, "Identification of the hub genes of periodontitis and diabetes by bioinformatics and experiment," has undergone thorough revisions, and I appreciate the authors' efforts in addressing the reviewers' feedback meticulously. The modifications significantly enhance the clarity and rigor of the study, particularly with the added flowchart, detailed descriptions of the methods and results, and improved figure readability. The authors have also provided a well-structured discussion, including the proposed future directions and acknowledgment of research limitations, making the study more robust. Moreover, the inclusion of computational models and ODE-based theoretical modeling studies as references aligns well with the study's objectives and brings additional depth to the discussion.

**Reply**: Thank you for your recognition.
